# Interferon-λ-neutralizing autoantibodies and common autoimmune disease autoantibodies in pediatric acute-onset neuropsychiatric syndrome

**DOI:** 10.3389/fimmu.2026.1832833

**Published:** 2026-07-17

**Authors:** Xihui Yin, Jennifer Frankovich, Muge Kalaycioglu, Ananya Choudhury, Michael J. Burry, Woo Joo Kwon, Lu Tian, Meiqian Ma, Bahare Farhadian, Cindy Manko, Melissa Silverman, Yuhuan Xie, Paula Tran, Noelle Schlenk, Paul J. Utz, Tyler R. Prestwood

**Affiliations:** 1 Division of Immunology and Rheumatology, Department of Medicine, Stanford University School of Medicine, Stanford, CA, United States; 2 Division of Allergy, Immunology, & Rheumatology, Department of Pediatrics, Stanford University School of Medicine, Stanford, CA, United States; 3 Stanford Immune Behavioral Health Clinic and Research Program, Stanford University & Stanford Children’s Health, Stanford, CA, United States; 4 Department of Biomedical Data Science, Stanford University School of Medicine, Stanford, CA, United States; 5 Division of Child & Adolescent Psychiatry, Department of Psychiatry and Behavioral Sciences, Stanford University School of Medicine, Stanford, CA, United States; 6 Institute for Immunity, Transplantation and Infection, Stanford University School of Medicine, Stanford, CA, United States; 7 Division of General Psychiatry and Psychology, Psychiatry Department of Psychiatry and Behavioral Sciences, Stanford University School of Medicine, Stanford, CA, United States

**Keywords:** autoantibody, autoimmunity, interferon-λ, pans, post-infection neuropsychiatric disorder

## Abstract

Pediatric Acute-onset Neuropsychiatric Syndrome (PANS) is characterized by sudden onset obsessive-compulsive disorder (OCD) symptoms in conjunction with other neuropsychiatric manifestations including disturbances in sleep, cognition, and behavior. Studies have revealed high rates of autoimmune and inflammatory markers—as well as comorbid rheumatologic disease—in patients with PANS. While published studies have suggested autoantibodies are common in PANS patients, the molecular targets of these autoantibodies (AAbs) remain poorly characterized. Here, we profile the AAbs in 224 plasma samples taken from 166 PANS patients during periods of active disease, or flares, compared to 83 pediatric healthy controls using custom Luminex microbead panels conjugated with highly curated antigens from common autoimmune diseases as well as cytokines and chemokines. We find that PANS patients exhibit increased prevalence of AAbs against autoantigens known to be targeted in scleroderma and GI/endocrine autoimmune conditions. Furthermore, a subset of PANS patients exhibited AAbs against IFN-λ, an important line of defense against infections at anatomic barriers. Among the 11 tested PANS plasma samples with IFN-λ-binding AAbs, 9 showed detectable inhibition of IFN-λ signaling, and 4 met our predefined stringent threshold for IFN-λ-neutralizing activity, while no tested HC samples met this neutralization threshold. These findings support a link between autoimmunity and PANS, and may provide insight into a potential disease mechanism mediated by immune deficits at barrier surfaces in a subset of patients.

## Introduction

Obsessive-compulsive disorder (OCD) affects 1-4% of children worldwide and typically exhibits a gradual onset, often beginning in early or late adolescence. In a subset of cases, OCD symptoms appear abruptly and are thought to be triggered by infection, commonly Streptococcal infection (1–7). Detailed clinical studies have led to the designation by expert panel consensus of Pediatric Autoimmune Neuropsychiatric Disorders Associated with Streptococcal infections (PANDAS) and the broader Pediatric Acute-onset Neuropsychiatric Syndrome (PANS) as subcategories of pediatric OCD (8, 9). In these cases, a post-infectious process driven by the immune system is strongly suspected.

Clinical evidence supports an autoimmune component of PANS. Sydenham chorea (SC)—a major neurological manifestation of acute rheumatic fever (ARF) marked by choreiform movements—is known to be triggered by streptococcal infections and anti-streptococcal antibodies that cross-react with central nervous system (CNS) antigens have been identified (10–12). Provided the symptom overlap between SC and PANS, we suspect that a similar mechanism may be contributing to disease activity in PANS. We have regularly observed subtle clinical markers of rheumatologic disease in PANS patients, including elevated levels of immune complexes, complement activation (low C4), enthesitis, arthritis, anemia, periungual redness and swelling (a finding in scleroderma and dermatomyositis), livedo reticularis, and other signs suggestive of small vessel inflammation (13, 14). PANS patients may also develop comorbid autoimmune conditions, including chronic arthritis, years after PANS onset (13). Furthermore, brain imaging studies, polysomnography studies (indicating movements during REM sleep), and neurological soft signs point towards a process in basal ganglia structures, much like what has been observed in SC and basal ganglia disorders including Parkinson’s disease (10, 15, 16). Many PANS patients also exhibit antinuclear antibodies (ANA). Increased antibody binding to mouse brain tissue has been observed using tissue sections incubated with PANS sera, particularly in brain regions implicated in OCD (17, 18), but specific AAb targets in PANS remain poorly characterized.

Increasing evidence points to infections as triggers for autoimmunity and immune dysfunction. Patients infected with SARS-CoV-2 or other viruses develop new-onset autoantibodies (AAbs) including anti-cytokine antibodies (ACA) with blocking activity, and multiple large epidemiologic studies have shown significantly increased prevalence of new AID in SARS-CoV-2-infected patients (19–22). Epstein Barr Virus (EBV) is similarly implicated in multiple sclerosis (MS) and systemic lupus erythematosus (SLE) (23–25). Collectively, these studies strongly suggest that bacterial and viral infections can trigger AID and CNS disease.

The relationship between infections and autoimmunity is bidirectional; in addition to infections triggering autoimmunity, self-directed antibody responses are also known to impact the course of infections. For example, pre-existing, type I interferon (IFN)-neutralizing AAbs have been associated with severe COVID-19, resulting in an estimated 20% of COVID-19-related deaths (26, 27). Type I IFN-neutralizing AAbs have also been associated with exacerbated viral encephalitis, influenza, and herpes virus infections, as well as an increased likelihood of recurrent infections (20, 28–32). Overall, these studies point to cytokine-neutralizing AAbs as determinants of disease severity.

Given this evidence, we hypothesize that AAbs may contribute to disease in a subset of patients with PANS. In the current study, we profile AAbs using plasma samples collected from a large cohort of patients with PANS taken during active disease and compare to healthy controls (HC). We utilize custom Luminex microbead-based panels consisting of highly-curated autoantigens from common AID, as well as cytokines, chemokines and growth factors. At the cohort level, AAb profiles between PANS patients and HCs revealed increased incidence of AAbs against autoantigens related to scleroderma/dermatomyositis and GI/endocrine autoimmunity. We also identify IFN-λ-binding AAbs in a subset of PANS patients. Although IFN-λ-neutralizing AAbs have been reported to be rare in the general population, several tested PANS samples demonstrated functional inhibition of IFN-λ signaling, with a subset meeting a stringent predefined threshold for neutralizing activity (33). Overall, these data provide further support for autoimmunity in PANS and identify a novel molecular pathway of potential importance in inflammatory CNS disease.

## Materials and methods

### Study cohort and selection criteria

The Stanford Immune Behavioral Health (IBH)/PANS Clinic is located in Menlo Park, California, and accepts patients from the surrounding counties. The IBH Clinic evaluated 458 consecutive patients who met clinic entry criteria between September 15, 2012, and January 6, 2023. Per protocol, all patients accepted for evaluation at the clinic were referred by their primary care provider and pre-screened to increase the probability of a PANS diagnosis. The pre-screening process includes a review of medical records and a screening questionnaire. Unclear cases are discussed with the patient’s primary doctor and other involved providers. Psychiatric diagnoses were classified by experienced pediatric psychiatrists (MT, MS, PT, YX). Of 458 patients considered for this study, 224 patients met PANS criteria, and 166 of these patients had at least one in-flare sample available and met other study criteria (**Supplementary Figure 1**). Several blood samples were collected from each patient (flare and recovery). Samples were excluded if the patient received IVIG (within 9 months) or Rituximab treatment (within 2 years) prior to collection. An additional flare sample was included if collected more than one year after the initial flare sample (maximum 2 in-flare samples per individual). The samples included in this study cohort (Illuminate 1a) will be included in further research studies (epitope mapping, further autoantibody arrays, proteomics, etc.). Healthy control (HC) samples (n = 83) were recruited from the same local youth community and were included if they had no known history of autoimmune or psychiatric condition, as well as no history of these conditions in a first-degree family member.

### Plasma samples

Plasma samples from PANS patients and controls were collected between September 19, 2013, and November 17, 2023. Aliquoted plasma samples were stored in the Stanford Biobank. Demographic and clinical information for these samples is described in detail below and in **Tables 1**–**4**. Three patients had onset of PANS after a SARS-CoV2 infection (PMID: 40174578).

**Table 1:** Count of blood specimens by type (flare v. recovery) from PANS subjects from the Illuminate 1 cohort.

Count of blood specimens by type (flare v. recovery) (Stanford Illuminate 1 Cohort)	PANS (n = 166)
First flare research draw captured in clinic	166 (100%)
Second flare research draw captured in clinic (>1 year after first flare)	59 (35%)
Best recovery research draw	98 (59%)

**Table 2:** Demographics of PANS subjects from the Illuminate 1 cohort and healthy controls.

Demographics of PANS cohort and healthy controls (Stanford Illuminate 1 Cohort)	PANS (n = 166)	Control (n = 83)
Age at disease onset, years (mean)	9.5 ± 3.7	–
Age at clinic presentation, years (mean)	10.6 ± 3.9	–
Age at first flare blood draw, years (mean)	11.5 ± 4.3	14.3 ± 5.0
Sex		
Male	101 (61%)	41 (49%)
Race		
White	138 (83%)	42 (51%)
Asian	5 (3%)	29 (35%)
Two or more races reported	22 (13%)	10 (12%)
Other	1 (<1%)	2 (2%)
Ethnicity		
Hispanic or Latino	23 (14%)	10 (12%)
County		
Resides within 13 surrounding counties	131 (79%)	83 (100%)

Data are presented in number (percentage) or mean ± SD unless specified.

**Table 3:** Symptoms at clinical presentation in PANS subjects from the Illuminate 1 cohort. OCD, obsessive compulsive disorder.

Symptoms at Clinic Presentation (Stanford Illuminate 1 Cohort)	PANS (n = 166)
Neuropsychiatric symptoms at clinic presentation, n (%)	
OCD	146 (88%)
Eating restriction (food refusal or avoidance)	82 (49%)
Anxiety	149 (90%)
Emotional lability and/or depression	144 (87%)
Irritability, aggression and/or severely oppositional behaviors	136 (82%)
Behavioral/developmental regression	76 (46%)
Cognitive impairment	85 (51%)
Sensory amplification	97 (58%)
Motor issues	63 (38%)
Urinary symptoms	70 (42%)
Sleep disturbances	113 (68%)
Flare status at clinic presentation	
In flare	136 (82%)
Not in flare	20 (12%)
Unclear flare/No data	10 (6%)
Psychometric scores at (or nearest) clinic presentation	
PANS Global Impairment Score (GIS) Range (0-100)^A,B^	47.9 ± 23.4
Caregiver Burden Inventory (CBI) Range (0-96)^A,C^	34.2 ± 18.0
Caregiver Burden Inventory (CBI) ≥ 36 ^C,D^	75 (47%)

Data are presented in number (percentage) or mean ± standard deviation.

A Higher scores indicate more impairment and burden for GIS and CBI.

B Of patients with available data (n=165).

C Of patients with available data (n=161).

D 36 is the threshold for needing respite care.

**Table 4:** Clinical findings.

Clinical findings	PANS (n = 166)
Any arthritis (meets ILAR and/or ASAS criteria) 50 (30%)	
Arthritis subtype (criteria met)	33 (20%)
Enthesitis-related arthritis (ILAR)	10 (6%)
Psoriatic arthritis (ILAR)	2 (1%)
Oligoarticular arthritis (ILAR)	25 (15%)
Spondyloarthritis (ASAS)	43 (26%)
Inflammatory back pain (Calin criteria)	24 (14%)
Any autoimmune/inflammatory disease (beyond arthritis)^A,B,C^	99/153 (65%)
Antinuclear antibody, titer ≥ 1:80	22/99 (22%)
Antihistone antibody, high	16/70 (23%)
Antithyroid antibody high ^C^	21/112 (19%)
C1q binding assay, high	25/120 (21%)
Complement 3, low	11/124 (9%)
Complement 4, low	41/126 (33%)
von Willebrand factor antigen, high	14/116 (12%)
D-dimer, high	6/111 (5%)
Physical examination findings (any)^D^	74/166 (45%)
Livedo reticularis, present	60/166 (36%)
Periungual redness, present	30/166 (18%)
Onychodermal band, abnormally prominent	54/166 (32%)

Data are presented in number (percentage) or mean ± SD unless specified.

A Some patients carry more than one diagnosis.

B These are thyroiditis (n=9), psoriasis (n=7), Celiac disease (n=3), chronic urticaria (n=3), primary immunodeficiency (n=2), inflammatory bowel disease (n=2), eosinophilic esophagitis (n=2), systemic lupus erythematosus (n=1), diabetes mellitus (n=1), period fever (n=1).

C Thyroglobulin antibodies and/or thyroid peroxidase antibodies.

D All the laboratory and physical examination signs presented were hypothesized to be relevant in the fall of 2012, and the clinicians have attempted to collect the data at the first flare captured in the clinic for all patients. Due to the severe psychiatric symptoms and young age of the study sample, we were unable to complete laboratory tests in all patients; denominators are reported for each marker.

Arthritis (according to ILAR and ASAS criteria) (34, 35), inflammatory back pain (according to Calin criteria) (36), autoimmune disease, and markers of inflammation at clinic presentation. ASAS, Assessment of SpondyloArthritis international Society; ILAR, International League of Associations for Rheumatology.

### Prototype autoantibody control samples

Positive control plasma samples for array experiments were derived from donors with prototype autoimmune disorders with known reactivity (topoisomerase 1 [Scl-70], centromere, Sjögren’s Syndrome type A [SSA], whole histones, mitochondria, thyroglobulin, and ribonucleoprotein [RNP]) and were purchased from ImmunoVision. Serum from patients with Autoimmune Polyglandular Syndrome Type 1 patients were generously provided by Dr. David Lewis (Stanford) and used for array experiments.

### Bead-based autoantigen arrays

Autoantigen arrays were generated and utilized as previously described (19). To construct the array, 1 × 10^6^ carboxylated magnetic beads per analyte (MagPlex-C, Luminex) were distributed into 96-well plates (Greiner BioOne), washed, and resuspended in phosphate buffer (0.1 M NaH_2_PO_4_; pH 6.2) using a plate magnet. Bead surfaces were activated by addition of 0.5 mg of 1-ethyl-3(3-dimethylamino-propyl)carbodiimide (Pierce) and 0.5 mg of N-hydroxysuccinimide (Pierce) according to Luminex protocol. After 20 minutes of activation, beads were washed into coupling buffer (0.05 M MES (2-(N-morpholino)ethanesulfonic acid); pH 5.0) and incubated with 8 μg of each analyte or control antibodies for 2 hours at room temperature. Conjugated beads were washed and resuspended in storage buffer [0.02% Tween 20 in phosphate-buffered saline (PBS-T), 0.1% BSA, and 0.05% sodium azide], and stored at -80 °C. Immobilization of the antigens was confirmed using the prototypical control human plasma samples with known reactivity as described above (Immunovision) or antibodies specific for 6× histidine epitope tags (Abcam, ab9108).

For array probing, plasma samples were diluted 1:100 in an assay buffer of 0.05% PBS-T supplemented with 1% (w/v) bovine serum albumin (BSA, Sigma-Aldrich) in 96-well plates. The bead array was distributed into a 384-well plate (Greiner) by transfer of 5 μL of bead array mixture per well, and then 45 μL of the diluted plasma were added into the 384-well plate. Samples were incubated for 60 min on a shaker at room temperature. Beads were washed three times with 60 μL of PBS-T on a plate washer (EL406, BioTek). Beads were resuspended in 50 μL of 1:1000 diluted R-phycoerythrin (PE)–conjugated Fcγ fragment–specific goat anti-human IgG (109-116-098, Jackson ImmunoResearch) in 0.05% PBS-T and incubated at room temperature for 30 min. Plates were washed three times with 60 μL of PBS-T and resuspended in 50 μL of PBS-T for analysis using a FlexMap3D instrument (Luminex Corporation). Binding events were displayed as Median Fluorescence Intensity (MFI). Duplicate plates were run for accuracy and quality control.

### IFN functional assays

IFN-λ functional assays were conducted using IFN-λ Reporter HEK 293 (HEK-Blue IFN-λ) cells (InvivoGen, hkb-ifnlv2). The commercial cell line, which is specific for type III IFN stimulation, contains stable overexpression of the human IFN-λ receptor (interferon lambda receptor 1 (IFNLR1) and interleukin-10 receptor 2 (IL10R2) chains) and secreted embryonic alkaline phosphatase (SEAP) under the control of the IFN-stimulated response element (ISRE). HEK-Blue IFN-λ cells were maintained and subcultured in growth medium supplemented with selective antibiotics following manufacturer’s instructions. For functional assays, HEK-Blue IFN-λ cells were seeded with IFN-λ into half-area 96-well plates at a density of 1.12 × 10^4^ cells per well without selection antibiotics and incubated for 24 hours. Each IFN-λ isoform was titrated using 3.16-fold serial dilutions. SEAP activity was measured with the data fitted to a Four Parameter Logistic (4PL) Curve using GraphPad Prism. The excitatory concentration required for 75% IFN-λ stimulation (EC_75_) was calculated using the following equation:


$$\log\left(EC_{50}\right)=\log\left(EC_{F}\right)-\left(\frac{1}{Hill\;Slope}\right)\times \log\left(\frac{F}{100-F}\right)$$

$$Y=Bottom+\frac{Top-Bottom}{1+10^{\log\left(EC_{50}-X\right)\times Hill\;Slope}}$$

where:

“Top” and “Bottom” are the maximum and minimum plateau values of the Y axis, respectively;
“Hill Slope” describes the steepness of the curve;
EC_50_ is the concentration of IFN-λ required to reach a SEAP readout halfway between the “Top” and “Bottom” values;
EC_F_ is the concentration of IFN-λ required to achieve F% of the SEAP readout between “Top” and “Bottom” values, where F = 75.

**Figure 1:**
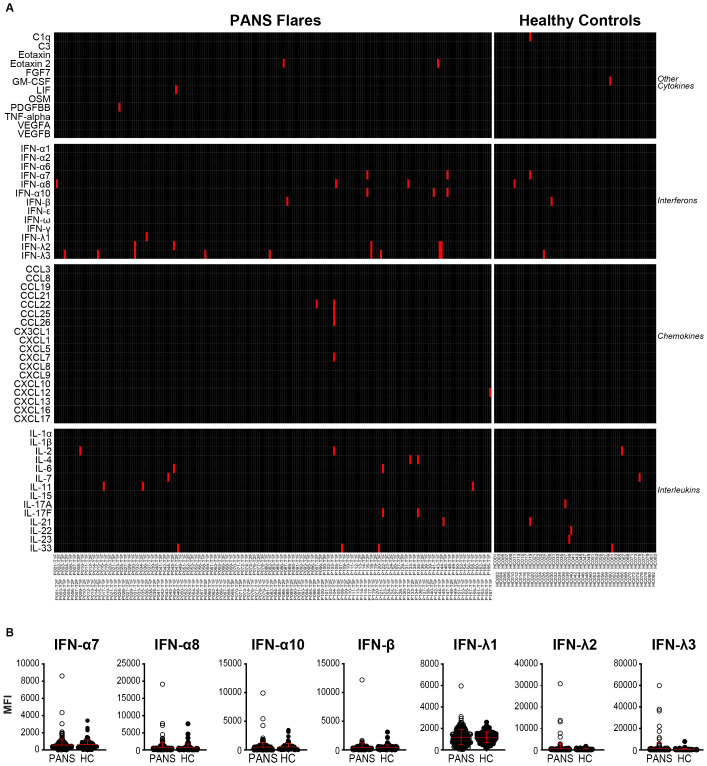
A subset of PANS patients harbors elevated anti-interferon autoantibodies. **(A)** Heatmap of the IgG autoantibodies detected using a 57-plex bead-based protein array. Individual antigens are shown in rows and labeled on the left. Autoantigen subpanels are grouped by immune molecule class, including IFNs, chemokines, interleukins and other cytokines, as labeled on the right. Samples are shown in columns and include PANS flares (left, n = 224) and healthy controls (HC, right, n = 83). Each cell represents plasma reactivity to a specific antigen in an individual sample. Samples meeting the positivity cutoff are shown in red, defined as both 5 SD above the mean HC MFI for that antigen and an absolute signal greater than 3,000 MFI. **(B)** Violin dot plots of specific antigens from the IFN panels showing many of the outliers with positive signal. Middle red lines denote the median MFI, while the lower and upper red lines correspond to the first and third quartiles, respectively.

To validate the IFN-λ neutralization assays, anti-IFN-λ monoclonal antibodies specific for each IFN-λ isoform were serially diluted 10-fold in growth medium and incubated for 30 minutes with the respective IFN-λ isoforms at their EC_75_ concentrations (IFN-λ1 = 0.5 ng/mL, IFN-λ2 = 1.1 ng/mL, IFN-λ3 = 1.4 ng/mL) before adding to cells (**Figure 1** and **S2**). To assess the neutralization activity of plasma, samples were heat-inactivated at 56 °C for 30 minutes. Plasma from patients or healthy control was diluted to 10% final concentration incubated for 30 minutes with IFN-λ at its EC_75_ concentration before addition to the freshly seeded HEK-Blue IFN-λ cells. Alternatively, purified plasma IgG and the corresponding IgG-depleted flow-through were diluted to match the IgG or plasma concentrations of the original samples. After 24 hours of stimulation, 20 µL of supernatant from each well was mixed with 180 µL of QUANTI-Blue™ solution (InvivoGen, rep-qbs) and incubated for 45 minutes. SEAP levels were measured by absorbance at 620 nm (OD 620) on a microplate reader (BioTek Synergy HT). Relative IFN-λ levels were determined by SEAP activity interpolated using a reference IFN-λ activity titration curve on the same plate. Activity values were normalized to control wells containing purified IFN-λ. The IFNLR1 neutralization assays were modified so that 10% plasma from patients or healthy controls was directly incubated with HEK-Blue IFN-λ cells, rather than with IFN-λ as above, for an hour before stimulation with IFN-λ1.

The vendors and catalog numbers for IFN-λ isoforms and their monoclonal antibodies are listed in **Supplementary Table 1**.

### IgG purification

A volume of 20 µL protein G magnetic resin (Lytic Solutions) was added to wells of a PCR plate and then washed with 100 µL PBS using a plate magnet. Resin was resuspended in 50 µL plasma diluted 1:1 with PBS. The plasma-resin mixture was incubated for 3h to overnight at 4°C on rotating mixer with inversion to maintain resin suspension. Samples were collected and subjected to subsequent rounds of incubation with magnetic resin until IgG levels became undetectable, as determined by ELISA described below. Resin with IgG bound was washed five times with 200 µL PBS and resuspended in 44 µL 100 mM glycine pH 2.4. This was incubated for 5 minutes at room temperature with periodic resuspension to elute IgG. The purified IgG in the supernatant fraction was collected, then 6 µL 1M Tris pH 8.0 was added to neutralize the solution.

### Quantification of human total IgG by ELISA

Human IgG concentrations were quantified using a human IgG ELISA Kit (Invitrogen™ 885055022) following the manufacturer’s instructions.

### Data processing and statistical analyses

Autoantigen array data processing, quality control and heatmap generation was carried out using R (4.2.3) and RStudio (RStudio Team, 2023.12.1). For quality control, wells with bead counts below 25 for any given antigen(s) were flagged. If the coefficient of variation (CV) of the replicate antigen MFI values was greater than 20% in the flagged well, the MFI value for that antigen was removed from analyses. Additionally, wells with positive-control antigen MFI values (human IgG from serum, anti-Human IgG Fc-fragment specific, anti-human IgG (H+L), anti-human IgG F(ab’) fragment-specific) that were below or above 2.5 standard deviation (SD) from the mean were also discarded from analysis. Finally, MFI values for unconjugated beads were subtracted from MFI values for antigen conjugated bead IDs, with MFI averages calculated after background subtraction. *ggplot2* (3.5.0) was used for heatmap generation.

Autoantibody positivity was defined using the following two criteria for determining antigen-specific thresholds. For each antigen, samples were considered positive only if the MFI exceeded the stringent cutoff of 5 SD above the HC mean (3 SD above the HC mean is a typically implemented threshold), thereby reducing the likelihood of false-positive calls in our exploratory arrays. The fixed 3000 MFI threshold was utilized to avoid falsely classifying low signals as positive for antigens with low background variance, as is consistent with our previously described work. Together, this combination of antigen specific and absolute signal floor MFI threshold facilitates the selection of samples with high autoreactivity for downstream functional neutralization assays (19, 37).

Binding AAb positivity for IFN-λ1, IFN-λ2, IFN-λ3, and IFNLR1 was defined using the same dual-threshold approach applied across the Luminex arrays. For each antigen, samples were considered binding-positive only if the MFI exceeded 5 SD above the HC mean for that antigen and exceeded an absolute signal threshold of 3,000 MFI. These thresholds were selected to prioritize high-specificity detection of strong autoreactivity in this exploratory research assay.

Neutralizing activity was defined based on inhibition of IFN-λ-induced signaling relative to the IFN-only/no-plasma condition. Samples were classified as neutralizing if residual IFN-λ-induced signaling was below 15% of the same-day median response in the median-binding HC comparator samples. This threshold was used as a conservative research definition of functional neutralization rather than a clinically validated diagnostic cutoff.

Statistical differences in AAb prevalence between patients and HCs for each subpanel were determined using two-sided Fisher’s exact test with Benjamini-Hochberg FDR corrections. Q values less than 0.05 were considered significant. Additionally, associations between clinical symptom outcomes and the presence of each Connective Tissue Disease (CTD) subpanel (predictor) were evaluated using chi-square tests of independence. P-values were calculated in Python 3 using the *SciPy* statistical library. For data visualization of violin dot plots, processed datasets were imported into *GraphPad Prism* (10.3.1).

### Data and code availability

Deidentified array data is publicly available on the Gene Expression Omnibus (GEO) database. The accession code is provided in our data availability statement.

## Results

### Demographic parameters of *Illuminate* 1 cohort

The course of PANS is characterized by a relapsing-remitting pattern, marked by flares (sudden exacerbations of psychiatric symptoms), followed by intervals of relative symptom stability or remission (38, 39). Patients (n = 166, flare draws = 224) had a median age (at first flare sample collection) of 11.3 years (x̄ = 11.5, s = 4.3, range 4-26). Controls (n = 83) had a median age (at sample collection) of 13.7 years (x̄ = 14.3, s = 5.0, range 4-24). Demographics of our patient and control cohorts are shown in **Table 2**. Across samples, we also compared demographic variables between the longitudinal PANS flare (n = 224) and HC cohorts (n = 83), as summarized in **Supplementary Table 6**. Overall, HCs were older than PANS patients (mean age 14.35 vs. 12.06 years; Welch’s t test p = 0.0003; median age 13.71 years vs. 11.70 years; Mann–Whitney U p = 0.0005). Sex distribution was not significantly different between groups, although there was a trend toward a higher proportion of males in the PANS cohort (60.3% vs. 48.2%; Fisher’s exact p = 0.069). Race/ethnicity differed significantly between groups (X^2^ p = 1.9 × 10^-13^), with a higher proportion of White participants in the PANS cohort (77.7% vs. 47.0%; Fisher’s exact p = 6.5 × 10^-7^) and a higher proportion of Asian participants in the HC cohort (33.7% vs. 2.7%; Fisher’s exact p = 9.5 × 10^-13^). Hispanic/Latino and Multiracial/Other representation did not differ significantly between groups. These demographic differences were considered when interpreting group-level AAb comparisons; however, limited subgroup sizes reduced power for robust multivariable adjustment across individual AAbs, AAb subpanels, IFN-λ-neutralizing AAbs, and detailed clinical phenotypes.

### PANS patients show clinical signs of autoimmunity

As part of clinical evaluation and treatment, patients undergo detailed clinical examination and laboratory workup. Clinical examination commonly demonstrates stigmata of autoimmune disease including arthritis, enthesitis, high levels of immune complexes, low C4, and periungual redness and swelling (**Table 4**).

### A subset of the PANS *Illuminate* 1 cohort exhibit high levels of AAbs associated with scleroderma and GI/endocrine autoimmunity

Approximately 1/4 of PANS patients exhibit anti-nuclear antibodies (ANA). Despite these observations, the autoantibody repertoire in PANS has not been characterized in detail. Therefore, we sought to evaluate the prevalence of AAbs characteristic of traditional autoimmune disease during PANS flares. To assess the breadth of AAbs targeting common autoantigens in connective tissue disease (CTD), we implemented a custom 27-plex traditional autoantigen array made up of highly curated antigens validated through previous studies (19, 20). The autoimmune diseases represented by these antigen panels include systemic lupus erythematosus (SLE), Sjögren syndrome, dermatomyositis, scleroderma, primary biliary cirrhosis, thyroiditis, pernicious anemia, and autoimmune vasculitides. A panel of prototype sera with known reactivity to several of these antigens served as positive controls.

**Figure 2:**
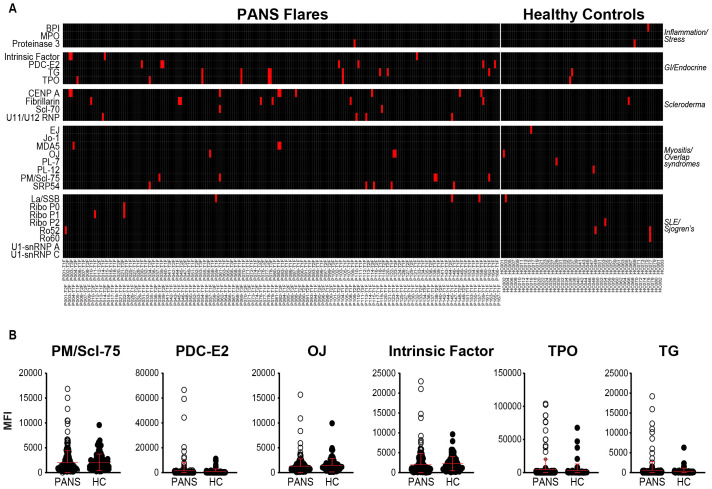
PANS patients demonstrate increased autoantibodies associated with scleroderma and GI/endocrine diseases. **(A)** Heatmap of IgG autoantibodies detected using a 27-plex bead-based protein array. Individual antigens are shown in rows and labeled on the left. Autoantigen subpanels are grouped according to associations with autoimmune diseases, tissue inflammation or stress responses, as labeled on the right. Samples screened are shown in columns and include PANS flare samples (left, n = 224), healthy controls (HC, right, n = 83). Each cell represents plasma reactivity to a specific antigen in an individual sample. Samples meeting the positivity cutoff are shown in red, defined as both 5 SD above the mean HC MFI for that antigen and an absolute signal greater than 3,000 MFI. **(B)** Violin dot plots of specific antigens from the scleroderma and GI/endocrine panels showing outliers with positive signal. Middle red lines denote the median MFI, while the lower and upper red lines correspond to the first and third quartiles, respectively.

Using a threshold of 5 SDs above the mean HC median fluorescence intensity (MFI) and a 3,000 MFI static cutoff, we assessed the prevalence of AAbs to these targets (**Figure 2A**). We observed several “hits” in the PANS group. To evaluate whether the presence of AAbs within each CTD subpanel in patients (predictor) was associated with the corresponding relevant clinical manifestations (outcomes), we applied chi-square tests of independence. To assess potential confounding by patient demographics, we tested for associations between demographic variables and the presence of CTD autoantibodies within each subpanel and found no significant associations (**Supplementary Tables 2–3**). We also did not observe significant associations between subpanel CTD AAbs and their respective clinical features. Specifically, the presence of scleroderma/myositis AAbs in patients were not associated with vascular blood markers (p = 0.67) or periungual redness swelling (p = 0.44). There was no association between GI/endocrine AAbs and the presence of prominent onychodermal bands (p = 0.96).

Given the small sample sizes for individual clinical variables tested, we further consolidated the subpanels and tested for association between the presence of any CTD AAbs and broader clinical outcomes. No associations were found between AAbs presence and flare duration (p = 0.86), arthritis (p = 0.38), or signs of vasculopathy (p = 0.49). On the other hand, the presence of other autoimmune conditions in PANS patients demonstrated a significant association with the presence of any CTD AAbs (p = 0.03) (**Table 5**). AAb positivity remained positively associated with presence of other immune-mediated conditions after adjusting for age, sex and race (p = 0.0210; Odds Ratio: 2.864; 95% CI: 1.172-6.997). Together, only coexisting autoimmune diseases were associated with CTD AAb positivity, likely reflecting the clinical heterogeneity of PANS and limited subgroup sizes, which reduced power to detect additional associations; at the same time, this finding is consistent with prior reports of increased personal and family history of autoimmune disease in PANS and suggests that CTD AAbs in a subset of patients may reflect broader immune dysregulation (**Table 4**).

**Table 5:** Relationship between clinical symptoms and CTD AAb status.

Variable	CTD AAbs present (n = 47)	CTD AAbs absent (n = 119)	p-value
Demographics (Control)			
Age at onset (years, average ± SD)	7.7 ± 3.5	8.2 ± 3.5	0.43
Sex (N male, %)	27 (57%)	74 (62%)	0.70
Race/Ethnicity (N non-Hispanic white, %)	34 (72%)	84 (71%)	0.97
Clinical symptoms (Outcomes)			
Duration of first flare (months)^A^			
*Average*	10.3	10.9	0.86
*SD*	17.7	16.6	
*Range*	[0.2, 107.8]	[0.2, 101.4]	
Arthritis (N, %)	17 (36%)	33 (28%)	0.38
Other autoimmune condition (N, %)	12 (26%)	13 (11%)	0.03
Vasculopathy (skin or blood marker) (N, %)	26 (55%)	57 (48%)	0.49

A Calculated out of the number of patients in each category with available data (N = 44/47; N = 114/119).

To compare the prevalence of AAbs in PANS patients relative to HC, we conducted Fisher’s exact test for AAbs in the scleroderma and GI/endocrine panels, both of which revealed significance (p = 0.009 and Benjamini-Hochberg q = 0.04; p = 0.0161 and Benjamini-Hochberg q = 0.04; **Supplementary Tables 4–5**). The scleroderma panel included CENP A (Centromere Protein A), Fibrillarin (U3 small nucleolar ribonucleoprotein), Scl-70 (topoisomerase I) and U11/12 RNP (ribonucleoprotein). The GI/endocrine panel included PDC-E2 (pyruvate dehydrogenase complex E2, a mitochondrial antigen targeted in primary biliary cirrhosis), thyroid antigens, and intrinsic factor (pernicious anemia) (**Figure 2B**). While the prevalence across the myositis/overlap syndrome panel did not differ statistically between PANS flares and HC (p = 0.62, Benjamini-Hochberg q = 0.62), several autoantigens including MDA5 (an antiviral, RNA-binding self-protein targeted in myositis), OJ (a tRNA synthetase target in myositis), and PM-Scl75 (an exosome protein autoantigen in overlap syndromes) exhibited a trend towards increased AAb prevalence and MFI values in PANS flare samples compared with HC. Of note, PANS flare samples did not exhibit an increased level of autoantibody binding against β-tubulin or GM1 ganglioside, which are previously described targets in SC (**Supplementary Figure 2**). Overall, the differences in AAb levels observed in PANS were due to an increased prevalence of AAbs in the specific AID subpanels described above, rather than group differences in mean AAbs levels, which were not statistically significant.

### A subset of patients with PANS produce AAbs that bind to type I and type III IFNs

Anti-cytokine AAbs (ACA) are increasingly recognized as important modulators of disease induced by infections, most recently with COVID-19 disease severity and anti-type I IFN AAbs (19, 26, 40). Given the post-infectious nature of PANS, we characterized the ACA repertoire in PANS patients and HCs using a custom 57-plex panel composed of 12 IFN molecules, 14 interleukins, 18 chemokines, and 12 other cytokines. Several individual patients exhibited high levels of AAbs against type I and type III IFNs, but we did not detect overall group differences in mean AAb levels (**Figure 1A** and **Supplementary Table 5**). We detected outlier patients with AAbs against IFN-α7, IFN-α8, IFN-α10, and IFN-β, as well as a larger, non-overlapping group producing AAbs against IFN-λ1, IFN-λ2 and IFN-λ3 (**Figure 1B**).

### Developing SEAP-based IFN neutralization assays

**Figure 3:**
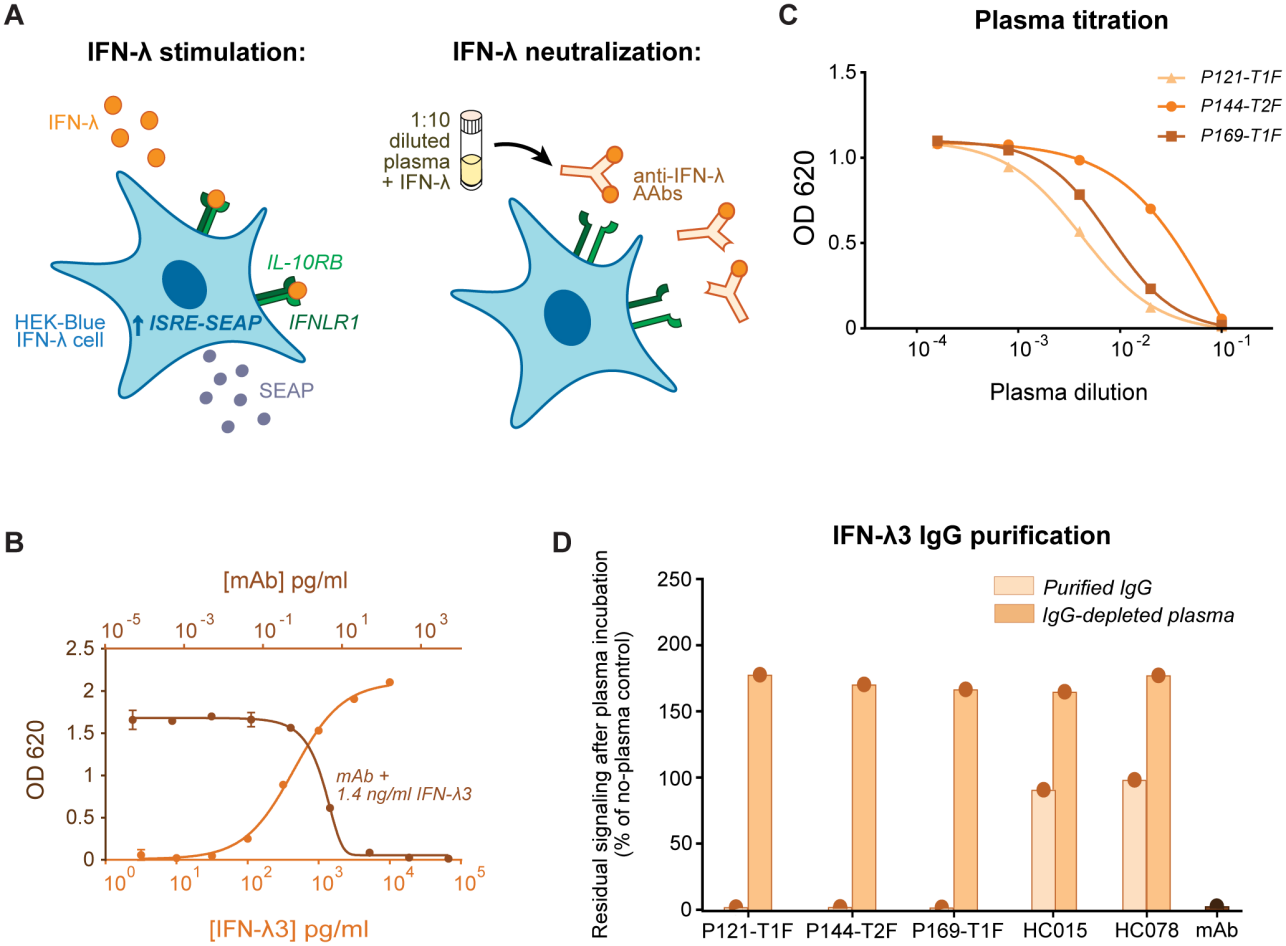
Selected patients exhibit potent Type III IFN neutralizing activity. **(A)** Schematic overview of the IFN-λ neutralization assay using HEK-Blue IFN-λ cells. Cells contain an ISRE-SEAP reporter system specific for Type III IFN stimulation. IFN-λ is incubated with 10% plasma before addition to cells. **(B)** Validation of the ISRE-SEAP reporter system in HEK-Blue IFN-λ cells with serial dilutions of IFN-λ3. Also, dose-dependent neutralization of IFN-λ3 at the EC_75_ concentration of 1.4 ng/mL in the presence of serially diluted anti-IFN-λ3 antibody (5 x 10–^4^ to 5 x 10^3^ ng/mL) mAbs reflect sensitivity of the neutralization assay. **(C)** Neutralizing activity of patient plasma with fully neutralizing IFN-λ3 AAbs. Samples were serially diluted 5-fold prior to incubation with IFN-λ3 (1.4 ng/ml). **(D)** IFN-λ3 neutralization assays using purified IgG from samples with high neutralizing activity compared with IgG-depleted plasma from the same samples and HC. IFN-λ3 mAb (5 µg/mL) was included as a control. Samples were incubated with IFN-λ3 (1.4 ng/mL).

To assess the functional effects of the identified IFN-neutralizing AAbs, we developed assays utilizing HEK Blue reporter cell lines (InvivoGen, San Diego, CA, USA), which produce secreted embryonic alkaline phosphatase (SEAP) in response to stimulation with specific cytokines (**Figure 3A**). Using these cells, we first validated their capacity to detect IFNs over a range of concentrations (**Figure 3B**). Next, we assessed the ability of this assay to detect the presence of neutralizing antibodies. Using a fixed concentration of IFN at approximately the EC_75_ (75% maximal effective concentration), we performed titrations of purified, neutralizing monoclonal antibodies against each IFN species. For example, increasing concentrations of IFN-λ3 displayed on a logarithm transformed scale show a sigmoidal response of SEAP signal secreted by the HEK-Blue IFN-λ-reporter cells (**Figure 3B**, lighter line). A reverse sigmoid response is observed when the concentration of IFN-λ3 is held constant at the EC_75_ of 1.4 ng/mL and increasing levels of IFN-λ3-neutralizing antibody are incubated with the cytokine prior to exposure to the target cells (**Figure 3B**, darker line). Very similar results were obtained with IFN-λ1 with EC_75_ of 0.5 ng/mL and IFN-λ2 with EC_75_ of 1.1 ng/mL (**Supplementary Figure 3**).

### PANS patients exhibit neutralizing AAbs against IFN-λ

**Figure 4:**
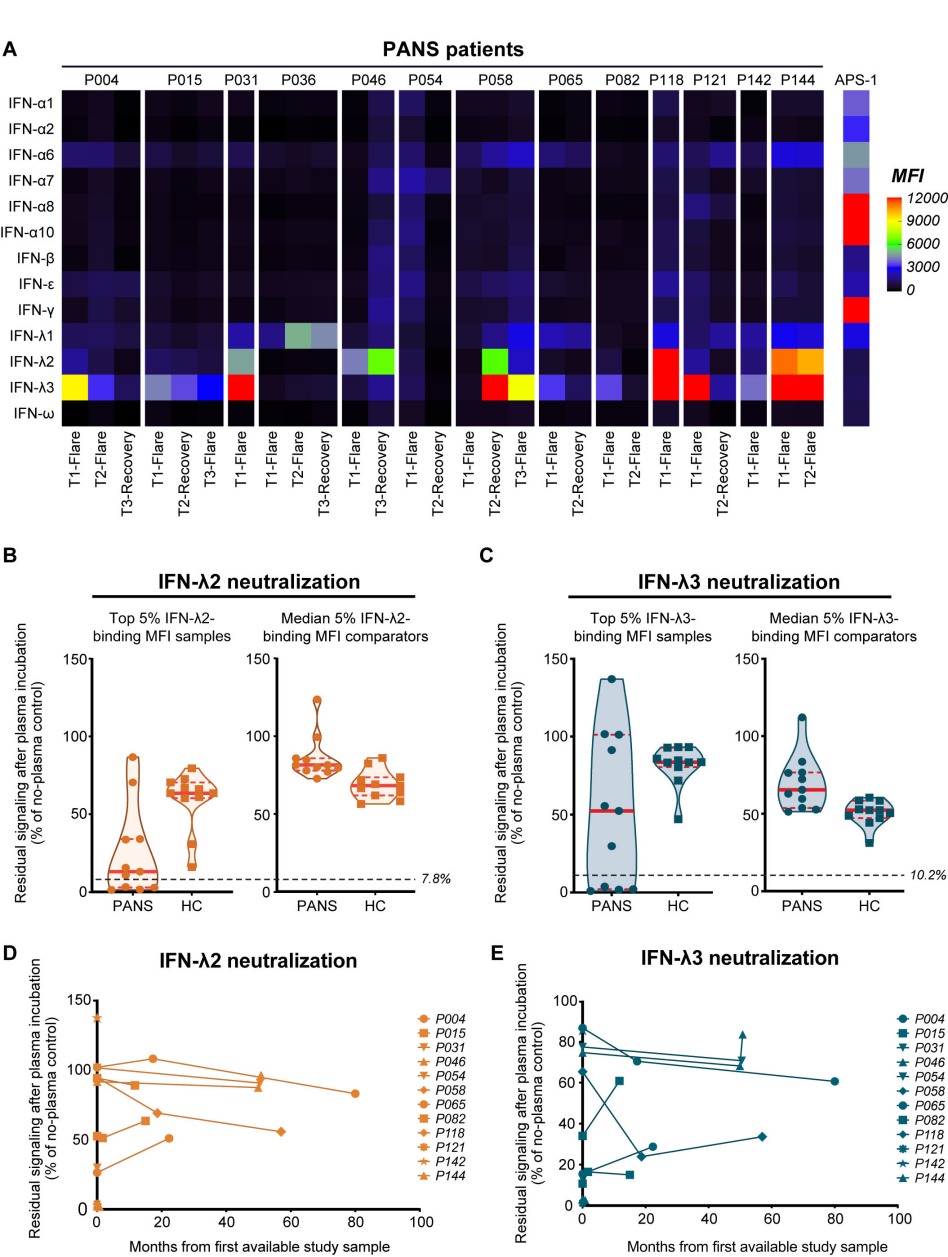
IFN-binding AAbs and IFN-λ neutralizing activity over time. **(A)** Heatmap showing anti-IFN IgG AAb binding in patients with high IFN-λ-binding reactivity and available longitudinal samples. Columns represent patient samples collected at the indicated study timepoints and clinical states. **(B–C)** Residual IFN-λ2 and IFN-λ3 signaling after plasma incubation in top 5% IFN-λ-binding MFI samples and median-binding comparator samples from PANS and HC groups. Lower residual signaling indicates greater IFN-λ-neutralizing activity. The dashed horizontal line indicates the predefined neutralization threshold. **(D–E)** Longitudinal IFN-λ2 and IFN-λ3 neutralization in patients with available serial samples. Samples are plotted according to months from the first available study sample, which does not necessarily correspond to initial PANS diagnosis, symptom onset, or first lifetime flare.

To determine whether the observed IFN-λ-binding AAbs in the PANS patient flare samples were functionally neutralizing, we sorted MFI reactivity values for IFN-λ2 and IFN-λ3 in descending order and selected the top 5% and median 5% of PANS flare samples (n = 11) to assess their neutralization potential (**Figure 1A** and **4B, C**). For comparison, we selected an analogous set of HC samples (n = 11). Because antigen binding and functional neutralization represent related but distinct measures of anti-IFN-λ autoreactivity, we classified IFN-λ pathway AAbs using separate criteria for binding positivity and neutralizing activity. Binding positivity was defined by antigen-specific MFI thresholds across the full cohort, whereas neutralizing activity was defined by functional inhibition of IFN-λ-induced signaling in the subset of samples selected for functional testing. The frequencies of IFN-λ1, IFN-λ2, IFN-λ3, and IFNLR1 binding AAbs, as well as IFN-λ2 and IFN-λ3 neutralizing activity, are summarized in **Table 6**. IFN-λ pathway-binding AAbs were identified in 14 PANS samples from 11 unique patients compared with one HC sample, but this difference did not reach statistical significance (p = 0.078). Functional neutralization was tested in high IFN-λ2 and IFN-λ3-binding samples and median-reactivity comparator samples from PANS and HC groups; therefore, neutralization frequencies are reported among tested samples and should not be interpreted as unbiased cohort-wide prevalence estimates. Among functionally tested samples, 4 PANS samples and no HC met criteria for IFN-λ pathway-neutralizing activity (**Table 6** and **Figures 4B, C**). In addition, among the 11 tested IFN-λ-binding PANS samples, 9 showed detectable inhibition of IFN-λ signaling, including the 4 samples that met the predefined stringent threshold for IFN-λ pathway-neutralizing activity. Similar to the prevalence of IFN-λ pathway-binding AAbs, the prevalence of IFN-λ pathway-neutralizing activity did not differ statistically between the selected PANS flares and HC samples (p = 0.09), despite a few PANS samples exhibiting potent neutralization capacity. Aside from IFN-λ2 and IFN-λ3, we also tested for functional inhibition of IFN-λ1 by samples with IFN-λ1-binding AAbs. We observed that a single PANS patient demonstrated detectable inhibition of IFN-λ1 signaling in our neutralization assay. However, because IFN-λ1 testing was not performed with a predefined comparator set as with IFN-λ2 and IFN-λ3, we could not determine whether this sample met the predefined criteria for neutralizing activity (**Supplementary Figure 4**).

**Table 6:** Frequency of IFN-λ/IFNLR1 binding and neutralizing autoantibodies in unique PANS and healthy control samples.

Measurement	Positivity definition	PANS, n/N (%)	HC, n/N (%)	Fisher’s exact p-value
IFN-λ1 binding AAb	MFI > 5 SD above HC mean and >3,000 MFI	1/224 (0.45%)	0/83 (0%)	1
IFN-λ2 binding AAb		5/224 (2.23%)	0/83 (0%)	0.3287
IFN-λ3 binding AAb		9/224 (4.02%)	1/83 (1.20%)	0.2972
IFNLR1 binding AAb		3/224 (1.34%)	0/83 (0%)	0.5659
Any IFN-λ ligand binding Aab	IFN-λ binding-positive	11/224 (4.91%)	1/83 (1.20%)	0.1915
Any IFN-λ pathway binding Aab	IFN-λ and/or IFNLR1 binding-positive	14/224 (6.25%)	1/83 (1.20%)	0.0782
IFN-λ2 neutralizing activity	Residual signaling<15% of median response in median-binding HC comparator samples	4/11 tested (36.36%)	0/11 tested (0%)	0.0902
IFN-λ3 neutralizing activity		4/11 tested (36.36%)	0/11 tested (0%)	0.0902
Any IFN-λ pathway-neutralizing activity	IFN-λ2 and/or IFN-λ3 neutralization	4/11 tested (36.36%)	0/11 tested (0%)	0.0902

Binding AAbs were measured across the full cohort. Functional neutralization assays were performed on a prioritized subset of samples selected for high IFN-λ/IFNLR1 binding reactivity, together with median-reactivity comparator samples from PANS and HC groups. Because the tested subset was enriched for candidate AAb-positive samples, neutralization frequencies are reported among tested samples and should not be interpreted as unbiased cohort-wide prevalence estimates.

To further characterize the neutralizing capacity of the PANS samples, we serially diluted (from 10% to 1%) the 3 PANS samples with highest binding to IFN-λ3 (**Figure 1A**). We observed potent neutralization of IFN-λ3 in 10% plasma, with more variable neutralization at 1% plasma and almost no neutralization at 0.1% plasma (**Figure 3C**). We isolated IgG with Protein A/G resin and compared the neutralization activity against IgG-depleted plasma in the neutralization assay to confirm that the IgG fraction—not the IgG-depleted plasma—exhibited the neutralizing activity (**Figure 3D**).

Finally, to examine the neutralization potential against other IFN species in which binding was observed in PANS flare samples, we performed neutralization assays using IFN-α7, IFN-α8, IFN-α10, and IFN-β on the samples identified in **Figure 1A** (data not shown). No neutralization was observed in any assay, demonstrating selective association between PANS and neutralizing AAbs against type III IFN but no other IFN types.

### IFN-binding AAbs are dynamic in PANS patients across time but do not correlate with disease states

To assess whether the levels of IFN-λ-binding AAbs are correlated with PANS disease activity, we analyzed AAb-binding to a panel of IFNs using samples from patients with IFN-λ-binding collected at additional timepoints including during periods of symptom improvement or recovery (**Figure 4A**). We found that levels of AAbs did not clearly correlate with disease state, as several patients exhibited the highest levels of IFN-λ-binding AAbs during their disease flares, while others exhibited the highest AAb levels during periods of recovery. Overall, these data demonstrate the temporal variability of AAb levels in PANS patients, as well as a lack of association with disease state.

For samples with sufficient remaining volume, we examined the neutralization potential of longitudinal samples collected over the months following the first available study sample (**Figures 4D–E** and **Supplementary Table 7**). For these analyses, the first available study sample did not necessarily correspond to initial PANS diagnosis, first symptom onset, or first lifetime flare. In several patients, the first available research sample was collected during a subsequent flare. We therefore label this as the first available study timepoint rather than disease onset. Neutralization capacity of plasma was not clearly predictable based on levels of IFN-λ-binding AAbs. Some patients exhibited similar levels of AAbs-binding to one IFN-λ subtype, but differing levels of neutralization. Overall, while the subtype specificity of IFN-λ-neutralizing AAbs was heterogeneous, neutralizing capacity was present in most patients with IFN-λ-binding AAbs.

**Figure 5:**
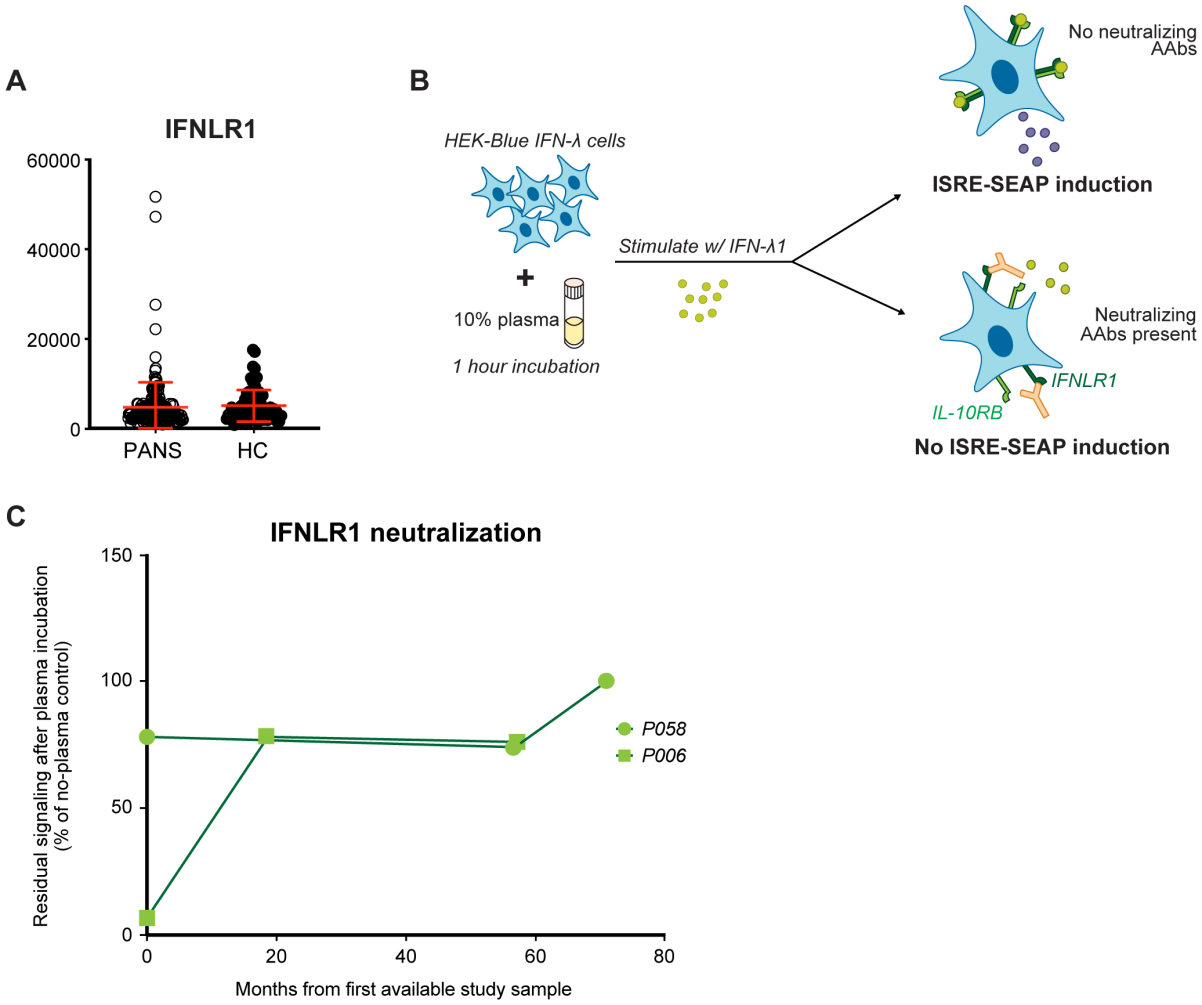
Anti-IFNLR1 autoantibodies in patients without type III IFN autoantibodies. **(A)** Violin dot plots of IFNLR1 antibody binding showing outliers with positive signal. Middle red lines denote the median MFI, while the lower and upper red lines correspond to the first and third quartiles, respectively. **(B)** Schematic overview of the IFNLR1 neutralization assay using HEK-Blue IFN-λ cells. Cells contain an ISRE-SEAP reporter system specific for Type III IFN stimulation. Cells are incubated with 10% plasma before addition of IFN-λ1 to cells. **(C)** Longitudinal IFN-λ1 inhibition in patients with positive IFNLR1 binding AAbs. Samples are plotted according to months from the first available study sample, which does not necessarily correspond to initial PANS diagnosis, symptom onset, or first lifetime flare.

Given that multiple patients exhibited neutralizing AAbs against IFN-λ targets (**Figures 3**-**4** and **Supplementary Figure 4**), we next evaluated the prevalence of AAbs against interferon lambda receptor 1 (IFNLR1), which determines responsiveness to IFN-λ, via Luminex microbead assay with IFNLR1 coupled directly to beads. We identified several PANS patients as outliers with much higher AAbs binding compared to HCs (**Figure 5A**). We next modified the SEAP-based IFN neutralization assay above to measure effects on cell surface receptors by pre-incubating diluted plasma with cells prior to cytokine exposure (**Figure 5B**). Using the samples which exhibited binding only to IFNLR1 (and not to IFN-λ1), we measured the capacity of diluted plasma to block IFN-λ1 activity on target cells longitudinally and across disease states. One sample demonstrated detectable inhibition of IFNLR1 during a disease flare, demonstrating that AAbs have the capacity to block signaling not just by binding to the ligand, but also to its cognate cell surface receptor (**Figure 5C** and **Supplementary Table 7**).

## Discussion

Here, we characterize the AAb repertoire against common autoantigens in PANS patients and highlight findings with potential clinical relevance, keeping in mind the need for validation of these findings in larger cohorts.

We found that AAbs associated with scleroderma and GI/endocrine autoimmune diseases have increased prevalence in PANS patients relative to healthy controls. These findings agree with the PANS clinical examination findings of periungual erythema, which is a classic finding associated with scleroderma and dermatomyositis (41). The etiologies of scleroderma and dermatomyositis remain unknown, though both demonstrate post-infectious subgroups, as well as subgroups in the paraneoplastic setting. In scleroderma, a cross-reactive, self-reactive immune response is believed to follow environmental exposures or infections including CMV, EBV and Parvovirus B19 (42–46). PANS does not have a known neoplastic association but is suspected to have a post-infectious component.

We also describe a novel association of IFN-λ-neutralizing AAbs with PANS. Type III IFN AAbs were originally described in autoimmune polyendocrinopathy candidiasis ectodermal dystrophy (APECED or APS-1) (47, 48). However, few studies have assessed the impact of type III IFN AAbs. Type I IFNs AAbs have been of investigative interest in recent years, as they are determinants of severe disease in COVID-19 (26, 27). The prevalence and function of type III IFN AAbs in the general population and in COVID-19 has also been studied (26, 33). Based on the study by Vanker et. al, type III IFN AAbs are more prevalent in the general population than type I IFN AAbs (8.5% vs. 3.9%, respectively). Both AAbs show a similar increase with age. Approximately 2/3 of type III IFN AAbs show no neutralizing activity and there is no known association with severe COVID-19 outcomes (33). It has been speculated that type III IFN AAbs may have little functional impact, given the redundancies of type I and type III IFN signaling. In contrast to these observations, we report a low prevalence of type III IFN AAbs among HCs (0.4%) with an increase in PANS patients (2.2%), the majority of whom exhibit detectable inhibition (82%, 9/11), and 4/11 met a predefined stringent neutralization threshold. These type III IFN AAb MFIs indicate relatively high levels, in contrast to the low levels observed in APS-1 (47). It remains to be determined, however, whether the observed AAbs have relevance to PANS disease.

While type III IFN AAb binding MFI did not correlate with disease activity in our cohort, this does not exclude the possibility that these AAbs are biologically relevant in a subset of PANS patients. However, it does indicate that levels of circulating AAb alone does not serve as a biomarker of PANS disease activity in this cohort. In general, binding MFI and neutralizing activity should be interpreted as related but non-equivalent measures of anti-IFN-λ autoreactivity. Binding assays identify antigen-specific reactivity, whereas neutralization assays measure functional blockade of cytokine signaling. Prior studies of anti-type I IFN AAbs have shown that anti-interferon AAb levels can vary over time and that binding titers alone often do not fully capture functional neutralizing activity. For example, Shaw et al. demonstrated that plasma titers of type I IFN AAbs in critically ill COVID-19 patients are highly variable over time, with some patients retaining potent neutralizing activity despite titers declining to levels comparable to HCs (49). Similarly, Su et al. reported associations between relatively low AAb levels and certain clinical manifestations of post-acute sequelae of SARS-CoV-2 infections (50). These findings suggest that functional neutralizing activity, timing of sampling, tissue compartmentalization, and host context may be more informative than circulating titers alone. Therefore, the absence of a simple longitudinal relationship between IFN-λ binding MFI and clinical disease activity does not exclude biological relevance, but it does indicate that circulating binding levels alone are unlikely to serve as a straightforward disease activity biomarker in this cohort. Larger longitudinal studies incorporating both binding and functional neutralization assays will be needed to determine whether IFN-λ-neutralizing AAbs identify a biologically distinct subset of PANS patients.

In contrast to type I IFN—which acts systemically due to the ubiquitous expression of IFN-α/β receptor—IFNLR1 determines responsiveness to IFN-λ and exhibits expression limited to barrier interfaces (51). Studies have shown that loss of IFN-λ renders increased susceptibility to infection at barrier surfaces, including the lungs, respiratory tract, gastrointestinal tract, skin and blood-brain barrier (33, 52–55). In a mouse model of West Nile Virus encephalitis, IFN-λ was found to restrict viral invasion of the CNS by tightening the blood-brain barrier, preventing further dissemination of peripheral molecules into the brain (56). These observations may be of particular importance in PANS, where the blood-brain barrier is believed to be compromised in the amygdala and basal ganglia regions (15, 57). Studies of post-COVID neurological syndromes have demonstrated that autoantibody responses may be compartmentalized within the CNS, with evidence of CSF-restricted neural antigens-targeting autoantibodies that are undetectable in the periphery (58). Notably, monoclonal antibodies isolated from the CSF of one patient targeted both antiviral and antineural antigens, suggesting a potential link between antiviral immune responses, autoreactivity and infection-associated neurologic sequelae. In the context of PANS, future studies investigating type III IFN-neutralizing AAbs in paired peripheral blood and CSF are warranted to determine whether these AAbs are present intrathecally and to clarify their potential relationship to neuroinflammatory and post-infectious neurologic disease.

PANS patients who exhibited AAbs in general on the CTD arrays were more likely to have other autoimmune diseases. Two of the PANS patients with IFN-λ-binding AAbs also exhibited relatively rare AAbs against MDA5, while one had common thyroglobulin-binding AAbs. In dermatomyositis, anti-MDA5 AAbs are associated with the development of rapidly progressive interstitial lung disease (59). In these patients, no signs of lung disease were observed.

Given the high rate of comorbid autoimmune conditions in this patient population, it is a challenge to disentangle the independent contributions of AAbs described in this study, and we cannot draw definitive conclusions about the role of these AAbs in PANS pathogenesis or disease progression. Our observations demonstrate multiple PANS patients harbor IFN-λ-neutralizing AAbs; however, this finding was present in only a subset of PANS patients and requires validation in larger cohorts. However, given the relatively low abundance of AAbs in the pediatric population, and the clear association of type I IFN-neutralizing AAbs with increasing age (26, 27), the occurrence of IFN-λ-neutralizing AAbs in PANS may be of clinical interest. Phenotypically, patients with IFN-λ-neutralizing AAbs were clinically very similar to other PANS patients, and distinguishing features were not identified in this subgroup. Given the heterogeneous nature of PANS, larger cohorts with longitudinal clinical phenotyping and integration of immune, genetic and proteomic profiling will likely be necessary for identification of distinct subgroups based on symptomatology, laboratory evidence, and molecular features.

We additionally identified one patient with IFNLR1-neutralizing AAbs. IFNLR1-neutralizing AAbs have not, to our knowledge, been previously described. It is important to note that prior large-scale investigations have not identified type I IFN receptor AAbs. Furthermore, IFNLR1-neutralizing AAbs were observed in only a single patient, and therefore, we cannot infer any association with PANS.

The higher prevalence of arthritis and other autoimmune diseases in autoantibody-positive (AAb+) patients is an important consideration when interpreting the CTD-array findings. Because the CTD panel includes autoantigens classically associated with autoimmune diseases, these AAbs in PANS patients may reflect co-existing or emerging autoimmune disease, or a broader propensity toward loss of immune tolerance, rather than being specific to PANS itself. Thus, the AAbs identified here should not be interpreted as pathognomonic PANS biomarkers. Nevertheless, this finding remains biologically and clinically relevant, as these AAbs may identify a subgroup of PANS patients whose neuropsychiatric symptoms occur in the context of systemic immune dysregulation, which may represent subclinical or evolving autoimmunity. This phenomenon is well-documented in autoimmune conditions. For example, patients with Celiac disease exhibit a significantly higher incidence of Type 1 Diabetes (T1DM) and autoimmune thyroiditis compared to the general population (60, 61). In those cases, organ-specific AAbs (such as anti-TPO or anti-GAD65) often appear years before the clinical manifestation of the secondary disease, serving as markers of an underlying autoimmune state (62, 63). Larger and long-term observational studies are needed to clarify whether the AAbs we have identified in PANS represent markers of generalized susceptibility to autoimmunity, early autoimmune processes or features associated with distinct PANS patient phenotypes.

An important limitation of the present study is that we used targeted autoantigen panels rather than an untargeted discovery platform. Unbiased approaches such as KREX, REAP, HuProt, MIPSA or related proteome-scale technologies are needed to further map the autoantibody specificities in PANS. Follow up studies using untargeted and more comprehensive discovery approaches, including neural antigen-focused arrays to examine serum and CSF are underway. Nevertheless, the untargeted approach used here and functional characterization of selected findings allowed identification of IFN-λ-neutralizing AAbs. ﻿Future studies will aim to assess the impact of these IFN-λ-neutralizing AAbs on the blood-brain barrier in PANS, particularly regarding the susceptibility of the brain to infections and inflammation.

## Data Availability

CTD raw and normalized array data has been deposited in the Gene Expression Omnibus (GEO) database under accession number GSE329268. Remaining array data will be available as **Supplementary Data**. Code used for data analysis and figure generation are available from the corresponding authors upon reasonable request.
