# Supplementary figures and images for: Interferon-λ-neutralizing autoantibodies and common autoimmune disease autoantibodies in pediatric acute-onset neuropsychiatric syndrome
Source: Front Immunol. 2026 Jul 17;17:1832833. doi: 10.3389/fimmu.2026.1832833 (PMC13423868; doi:10.3389/fimmu.2026.1832833)

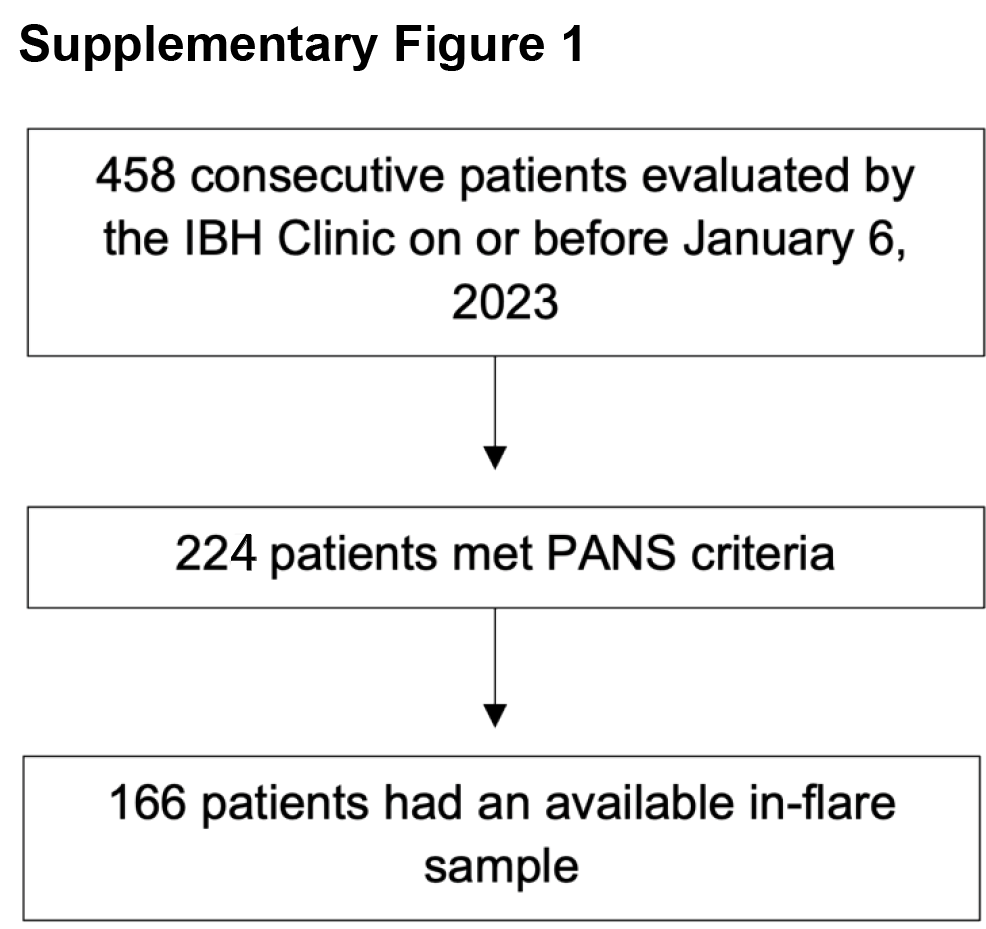

Supplement: Supplementary file 1 [file Image1.tif]

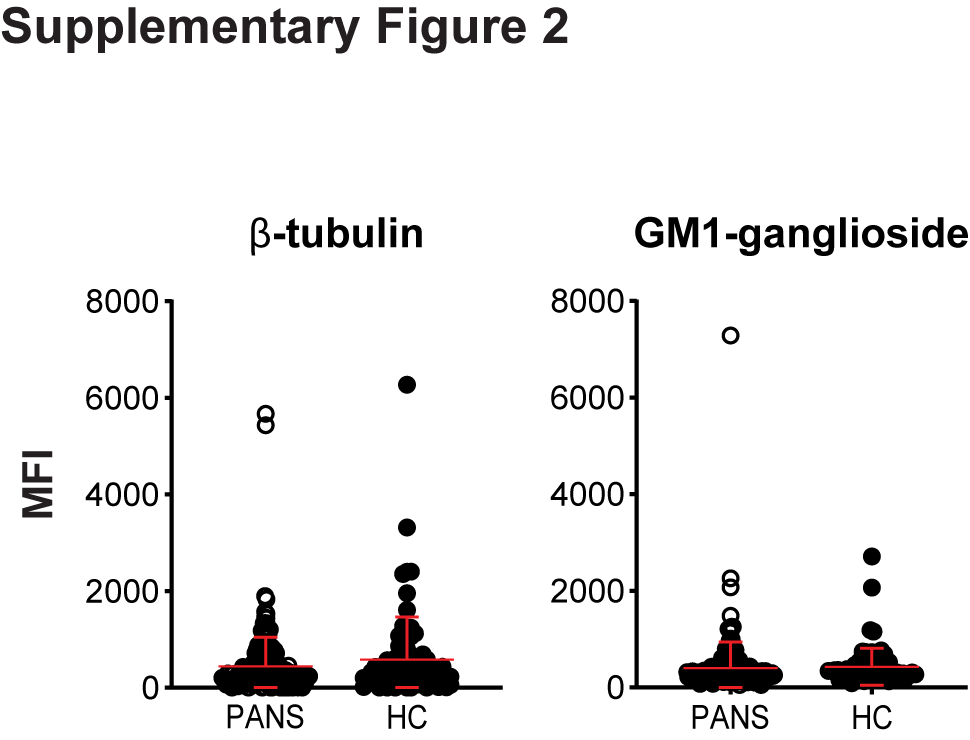

Supplement: Supplementary file 2 [file Image2.tif]

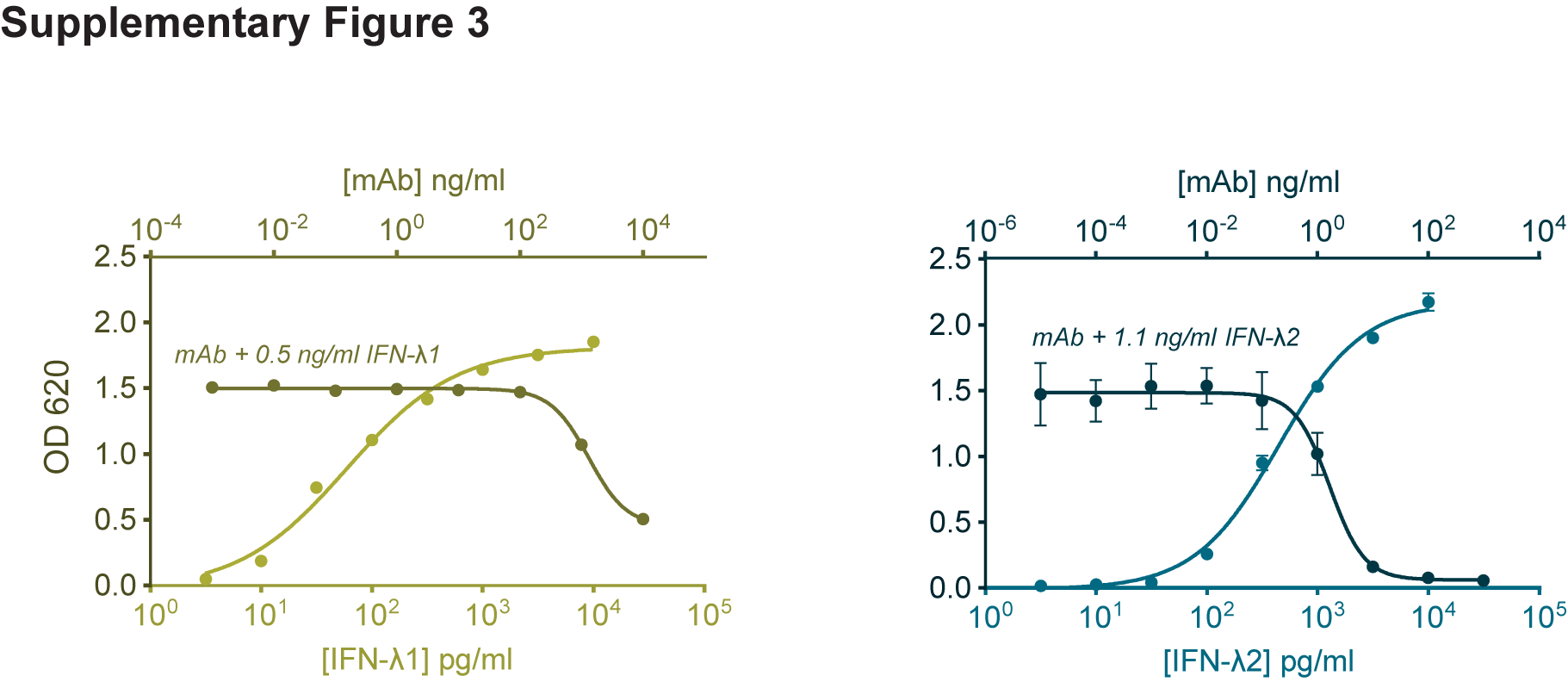

Supplement: Supplementary file 3 [file Image3.tif]

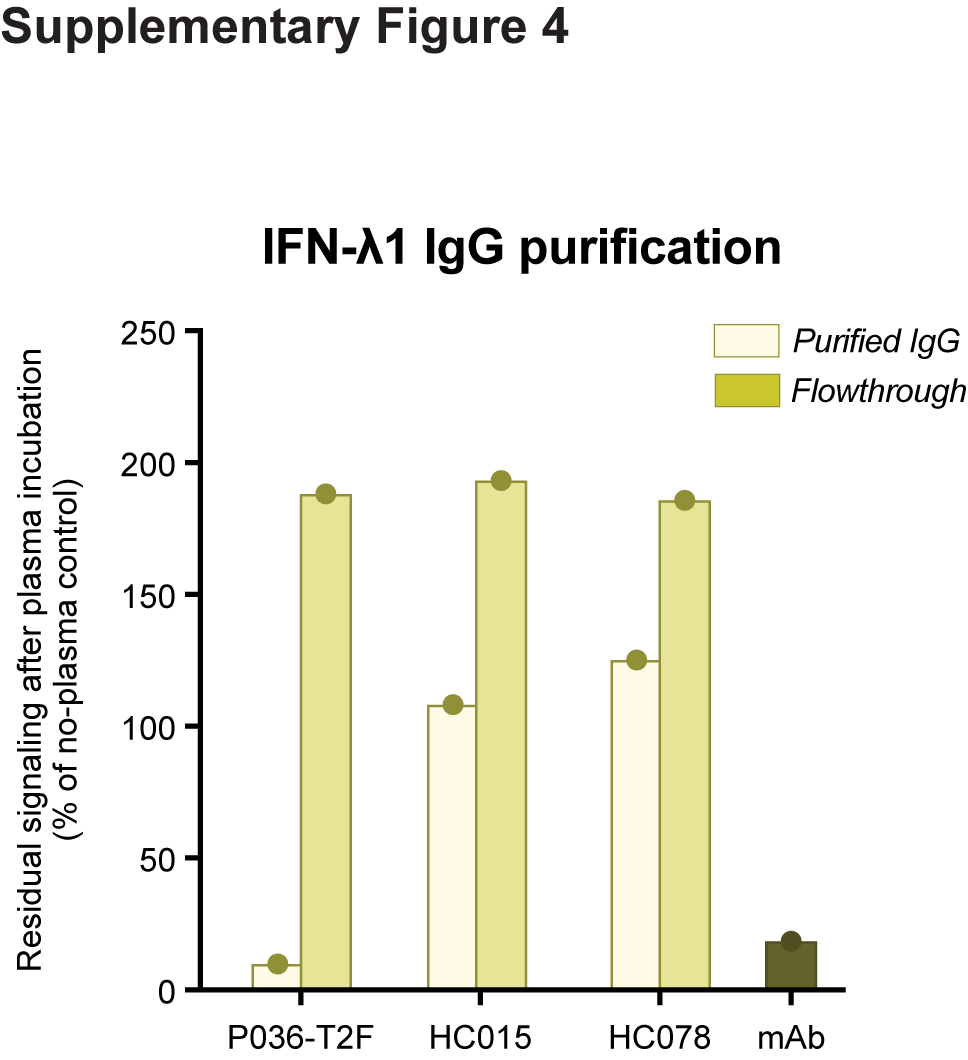

Supplement: Supplementary file 4 [file Image4.tif]
