# Supplementary material for: Interferon-λ-neutralizing autoantibodies and common autoimmune disease autoantibodies in pediatric acute-onset neuropsychiatric syndrome
Source: Front Immunol. 2026 Jul 17;17:1832833. doi: 10.3389/fimmu.2026.1832833 (PMC13423868; doi:10.3389/fimmu.2026.1832833)
Supplement: Supplementary file 5 [file Supplementaryfile1.docx]

Supplementary Material

## Supplementary Tables

**Supplementary Table 1.** *Catalog numbers for IFN-λ isoforms and their monoclonal antibodies.*

| **Product** | **Manufacturer** | **Catalog number** |
| --- | --- | --- |
| Interferon-λ1, recombinant human | Peprotech | 300-02L |
| Interferon-λ2, recombinant human | Peprotech | 300-02K |
| Interferon-λ3, recombinant human | PBL Assay Science | 11730-1 |
| Anti-Interferon-λ1 monoclonal antibody | InvivoGen | mabg-hil29-3 |
| Anti-Interferon-λ2 monoclonal antibody | InvivoGen | mabg-hil28a |
| Anti-Interferon-λ3 monoclonal antibody | InvivoGen | mabg-hil28b |

**Supplementary Table 2.** *Relationship between scleroderma/myositis AAb positivity and clinical markers of systemic inflammation.*

| **Variable** | **Scleroderma/Myositis AAbs present (n = 28)** | **Scleroderma/Myositis AAbs absent (n = 138)** | **p-value** |
| --- | --- | --- | --- |
| **Demographics (Control)** |  |  |  |
| Age at PANS onset (years, average ± SD) | 7.3 ± 3.2 | 8.2 ± 3.5 | 0.20 |
| Sex (N male, %) | 17 (61%) | 84 (61%) | 1.00 |
| Race/Ethnicity (N non-Hispanic white, %) | 20 (71%) | 98 (71%) | 1.00 |
| **Clinical symptoms (Outcomes)** |  |  |  |
| Vascular blood marker (N, %) | 4 (14%) | 13 (9%) | 0.67 |
| Periungual redness/swelling (N, %) | 7 (25%) | 23 (17%) | 0.44 |

**Supplementary Table 3.** *Relationship between GI/endocrine AAb positivity and presence of prominent onychodermal band.*

| **Variable** | **GI/Endo AAbs present (n = 18)** | **GI/Endo AAbs absent (n = 148)** | **p-value** |
| --- | --- | --- | --- |
| **Demographics (Control)** |  |  |  |
| Age at PANS onset (years, average ± SD) | 7.5 ± 3.9 | 8.1 ± 3.4 | 0.52 |
| Sex (N male, %) | 8 (44%) | 93 (63%) | 0.21 |
| Race/Ethnicity (N non-Hispanic white, %) | 13 (72%) | 105 (71%) | 1.00 |
| **Clinical symptoms (Outcomes)** |  |  |  |
| Prominent onychodermal band (N, %) | 7 (39%) | 52 (35%) | 0.96 |

**Supplementary Table 4.** *Fisher exact tests for the association between the prevalence of Connective Tissue Disease (CTD) subpanel AAbs and PANS disease.*

| **Subpanel** | **Fisher exact p-value** | **Benjamini-Hochberg (BH) q-value** | **Odds ratio** |
| --- | --- | --- | --- |
| Inflammation/Stress | 0.179462 | 0.299103 | 0.184054 |
| GI/Endocrine | 0.016124 | 0.040310* | 4.931034 |
| Scleroderma | 0.008864 | 0.040310* | 8.331808 |
| Myositis/Overlap syndromes | 0.617003 | 0.617003 | 1.486486 |
| SLE/Sjogen's | 0.325328 | 0.406660 | 0.516863 |

## * Denotes tests below BH q-value threshold of 0.05

**Supplementary Table 5.** *Fisher exact tests for the association between the prevalence of Cytokine & Chemokine (CC) subpanel AAbs and PANS disease.*

| **Subpanel** | **Fisher exact p-value** | **Benjamini-Hochberg (BH) q-value** | **Odds ratio** |
| --- | --- | --- | --- |
| Other cytokines | 1 | 1 | 0.922078 |
| Interferons | 0.140734 | 0.562937 | 2.233380 |
| Chemokines | 0.332704 | 0.665408 | Inf |
| Interleukins | 0.813910 | 1 | 0.846154 |

## * Denotes tests below BH q-value threshold of 0.05

**Supplementary Table 6.** *Comparisons of demographic variables between the longitudinal PANS flare and HC samples.*

| **Variable** | **PANS** | **HC** | **Statistical test** | **p value** | **Interpretation** |
| --- | --- | --- | --- | --- | --- |
| **N** | 224 | 83 | — | — | Final analyzed cohort |
| **Age, mean ± SD** | 12.06 ± 4.28 | 14.35 ± 5.00 | Welch’s  t-test | 0.0003 | HCs were older than PANS patients |
| **Age, median**  **[IQR]** | 11.70  [8.78–14.51] | 13.71  [10.38–18.20] | Mann–Whitney U | 0.0005 | HCs were older than PANS patients |
| **Male sex, n**  **(%)** | 135/224  (60.3%) | 40/83  (48.2%) | Fisher’s exact test | 0.069 | Not significantly different; trend toward more males in PANS |
| **White, n**  **(%)** | 174/224  (77.7%) | 39/83  (47.0%) | Fisher’s exact test | 6.5 x 10^-7^ | White participants were overrepresented in PANS |
| **Asian, n**  **(%)** | 6/224  (2.7%) | 28/83  (33.7%) | Fisher’s exact test | 9.5 x 10^-13^ | Asian participants were overrepresented in HCs |
| **Hispanic/Latino, n**  **(%)** | 34/224  (15.2%) | 10/83  (12.0%) | Fisher’s exact test | 0.584 | Not significantly different |
| **Multiracial/Other, n**  **(%)** | 10/224  (4.5%) | 6/83  (7.2%) | Fisher’s exact test | 0.386 | Not significantly different |
| **Overall race/ethnicity distribution** | Asian 2.7%  Hispanic/Latino 15.2%  Multiracial/Other 4.5%  White 77.7% | Asian 33.7%  Hispanic/Latino 12.0%  Multiracial/Other 7.2%  White 47.0% | Χ^2^ test | 1.9 x 10^-13^ | Significant racial/ethnic distribution differences |

**Supplementary Table 7.** *List of patients and their corresponding samples screened for IFN-λ pathway neutralization.*

| **Patient** | **Sample** | **Months from first sample collection** | **IFN-λ pathway binding AAb** |
| --- | --- | --- | --- |
| P004 | P004-T1F | 0 | IFN-λ3 |
|  | P004-T2F | 17.3 | - |
|  | P004-T3R | 80 | N/A |
| P006 | P006-T1F | 0 | IFNLR1 |
|  | P006-T2F | 18.7 |  |
|  | P006-T3R | 15 | N/A |
| P015 | P015-T1F | 0 | IFN-λ3 |
|  | P015-T2R | 1.7 | N/A |
|  | P015-T3F | 15 | - |
| P031 | P031-T1F | 0 | IFN-λ2  IFN-λ3 |
| P036 | P036-T1F | 0 | - |
|  | P036-T2F | 41.7 | IFN-λ1 |
|  | P036-T3R | 57.3 | N/A |
| P046 | P046-T1F | 0 | IFN-λ2 |
|  | P046-T2F | 49.9 | - |
|  | P046-T3R | 50.8 | N/A |
| P054 | P054-T1F | 0 | - |
|  | P054-T2R | 50.5 | N/A |
| P058 | P058-T1F | 0 | IFNLR1 |
|  | P058-T2R | 18.7 | N/A |
|  | P058-T3F | 57 | IFN-λ3 |
| P065 | P065-T1F | 0 | - |
|  | P065-T2R | 22.3 | N/A |
| P082 | P082-T1F | 0 | IFN-λ3 |
|  | P082-T2F | 11.7 | - |
| P118 | P118-T1F | 0 | IFN-λ2  IFN-λ3 |
| P121 | P121-T1F | 0 | IFN-λ3 |
| P142 | P142-T1F | 0 | - |
| P144 | P144-T1F | 0 | IFN-λ2  IFN-λ3 |

Patients and their corresponding samples positive for IFN-λ pathway-binding autoantibodies (AAbs) are shown along with the number of months elapsed since the first available study sample.

## Supplementary Figures

**Supplementary Figure 1.** *Flowchart of cohort subject selection process.*

**Supplementary Figure 2.** *Reactivity against Sydenham chorea autoantigens amongst PANS patients.* Violin dot plots of antigens associated with Sydenham chorea (β-tubulin and GM1-ganglioside). Middle red lines correspond to the median MFI, while the lower and upper red lines correspond to the first and third quartiles, respectively.

**Supplementary Figure 3.** *Validation of the ISRE-SEAP reporter system in HEK-Blue IFN-λ cells with serial dilutions of IFN-λ1 or IFN-λ2.* Dose-dependent neutralization of IFN-λ1 (0.5 ng/mL) or IFN-λ2 (1.1 ng/mL) at EC_75_ concentrations by anti-IFN-λ1 (10^-3^ to 10^4^ ng/mL) or anti-IFN-λ2 (10^-5^ to 10^3^ ng/mL) mAbs reflect sensitivity of the neutralization assay to various IFN-λ isoforms.

**Supplementary Figure 4.** *IFN-λ1 neutralization assay comparing purified IgG from patient plasma with IFN-λ1 binding AAbs, and healthy controls without binding AAbs.* 20% IgG-depleted flow-through plasma, which was 1:1 diluted during IgG purification, from each purified sample and IFN-λ1 mAbs (5 µg/mL) were included as controls. Samples were incubated with IFN-λ1 (0.5 ng/mL).
